# Allocating scarce medical resources during armed conflict: ethical issues

**DOI:** 10.1186/s40696-017-0033-z

**Published:** 2017-05-22

**Authors:** Nicholas Greig Evans, Mohamed A. Sekkarie

**Affiliations:** 1 University of Massachusetts Lowell, Lowell, MA USA; 2 Nephrology and Hypertension Associates, Bluefield, WV 24701 USA

**Keywords:** Peritoneal Dialysis, Healthcare Worker, Armed Conflict, Residual Renal Function, Allocation Principle

## Abstract

We describe ethical issues arising in the allocation of civilian medical resources during armed conflict. Three features are significant in the context of allocating scarce resources in armed conflicts: the distinction between continuous and binary medical resources; the risks of armed conflict itself, and the impact of cultural differences on cases of armed conflict. We use these factors to elicit a modified principle for allocating medical resources during armed conflict, using hemodialysis for patients with end-stage renal disease as a case study.

## Background

Armed conflict jeopardizes patient care, *inter alia,* through shortages in vital medical supplies. When healthcare resources are both scarce and insecure, ethically justified principles for resource allocation are required. These allocation decisions present a challenge for medical ethics in the context of armed conflict.

Existing statements on medicine and armed conflict tend to provide conflicting advice on allocation decisions. The *World Medical Association Regulations in Times of Armed Conflict and Other Situations of Violence* claims “[w]hether civilian or combatant, the sick and wounded must receive promptly the care they need. No distinction shall be made between patients except those based upon clinical need [1].” A joint statement by the International Commission of the Red Cross and Red Crescent, with advice by four other nongovernment organizations, states “in providing the best available care, [health care personnel] shall take into consideration the equitable use of resources [2],” but provides no guidance on what this equity might look like. These statements, however, ignore the realities of armed conflicts and resource scarcity. Clinical need is indeed a defensible principle on which to allocate resources, but it neither the only, nor always the most important principle for allocating scarce resources. Equity is another important principle in resource allocation, but what constitutes equality or equal treatment includes the possibility lotteries or “first come first serve” policies that carry with them numerous ethical and logistical issues [3].

While there exists a literature on allocation and treatment decisions for military healthcare workers and warfighters [4], little scholarship addresses with the ethics of allocating scarce medical resources to civilians operating within warzones. For example, in their description of moral dilemmas faced by staff at the Rambam Medical Center during the Second Lebanon War, Bar-El et al. [5] describe challenges faced in allocating fortified spaces to patients to protect them from potential rocket attacks, but give no guidance on how this challenge was resolved in a principled manner. Literature exists on allocating resources during mass casualty events such as disease pandemics or bioterror [6], and on medical rationing and triage decision for military doctors working with service personnel [7]. None of these, however, provide an account of the principled allocation of scarce medical resources to noncombatant civilians during armed conflicts.

In this article, we attempt to redress this neglect by describing general ethical considerations that govern the allocation of scarce medical resources within civilian contexts during armed conflict. We begin by identifying relevant ethical considerations that bear on this context, and how they differ from related situations in which allocation decisions must be made. We then apply our analysis to the illustrative case of hemodialysis among patients suffering from end-stage renal disease (ESRD) in the Syrian armed conflict, now in its seventh year.

## Resource allocation in armed conflict

In armed conflict, many if not most medical supplies—including healthcare workers—experience severe shortages through the destruction of trade routes, manufacturing and storage facilities, and hospitals. During World War One, it was estimated that 800,000 civilians died from a combination of material deprivation crowding and breakdowns in sanitary systems, and shortages of medical care. These deprivations, moreover, had long term effects on mortality for the survivors of war [8].

These shortages are of two kinds. First, shortages in medical supplies, combined with increases in demand for medical care due to civilian casualties of war cause severe *scarcity* among medical supplies: there simply aren’t enough to go around. Second, difficulties in providing aid to a warzone make the same resources radically *insecure*. Scarcity and insecurity create a situation where supplies must be allocated in an ethical manner: that is, allocation according a system of consistent and appropriate values.

Attempts to allocate scarce medical resources in an ethical manner date back to the first hemodialysis units [9], and have developed for a range of important scenarios that entail situations of scarcity and insecurity [3]. Solid organ are allocated on principles including equality and priority, factoring in the time a candidate is on waiting lists, their medical need, and prognosis. During mass casualty events, in contrast, allocation favors healthcare workers and other critical personnel, in an attempt to save the most lives and maximize the utility of scarce resources (Table 1).

**Table 1:** Monstic principles for allocating scarce resources. Adapted from Persad et al. [3]

Allocation principle	Advantages	Disadvantages	Examples of use
Treating people equally			
Lottery	Hard to corrupt; little information about recipients needed	Ignores other relevant principles	Military draft; schools; vaccination
First-come, first-served	Protects existing doctor-patient relationships; little information about recipients needed	Favors wealthy; powerful, and well-connected; ignores other relevant principles	Intensive Care Unit (ICU) beds; part of organ allocation
Favoring the worst-off: prioritiarianism			
Sickest first	Aids those who are suffering right now; appeals to “rule of rescue”; makes sense in temporary scarcity; proxy for being worst off overall	Surreptitious use of prognosis; ignores needs of those who will become sick in future; might falsely assume temporary scarcity; leads people receiving interventions only after prognosis deteriorates; ignores other relevant principles	Emergency rooms; part of organ allocation
Youngest first	Benefits those who have had least life; prudent planners have an interest in living to old age	Undesirable priority to infants over adolescent and young adults; ignores other relevant principles	New National Vaccine Advisory Committee/Advisory Committee on Immunization Practices (NVAC/ACIP) pandemic flu vaccine proposal
Maximizing total benefit: utilitarianism			
Number of lives saved	Saves more lives, benefiting the greatest number; avoids need for comparative judgments about quality or other aspects of lives	Ignores other relevant principles	Past ACIP/NVAC pandemic flu vaccine; bioterrorism response policy; disaster triage
Prognosis or life-years saved	Maximizes life-years produced	Ignores other relevant principles, particularly distribute principles	Penicillin allocation; traditional military triage (prognosis) and disaster triage (life-years saved)
Promoting and rewarding social usefulness			
Instrumental value	Helps promote other important values; future oriented	Vulnerable to abuse through choice of prioritized occupations or activities; can direct resources away from health needs	Past and current NVAC/ACIP pandemic flu vaccine policy
Reciprocity	Rewards those who implemented important values; past oriented	Vulnerable to abuse; can direct health consequences; intrusive assessment process	Some organ donation polices

Armed conflict share features with the above paradigms. Solid organs are both scarce and insecure resources: there aren’t enough for everyone, and no guarantee on when and where more will emerge [10]. Mass casualty events, on the other hand, share with armed conflict a systemic and widespread set of harms [6, 11]. There, considerations of societal utility—in particular, maintaining critical services in the face of extreme adversity—may outweigh other considerations. However, three features further distinguish armed conflict from other allocation principles.

## Continuous versus binary resources

War is a protracted disaster: the Syrian conflict is in its seventh year. Allocation principles must therefore account for long—potentially indefinite—periods of scarcity and insecurity. This will depend in part on whether resources are binary, or continuous. Binary resources are indivisible for the purpose of care, e.g., any attempt to transplant a fraction of a heart would waste that heart. Pain medication, on the other hand, could conceivably be divided during extended shortages to produce clinically meaningful if non-ideal outcomes. Some resources, such as antibiotics, may be divided only to a certain therapeutic threshold, after which subtherapeutic doses fail to provide meaningful clinical outcomes.

This distinction has implications for resource allocation in armed conflict. When continuous supplies are scarce, there is a case to be made for ensuring sufficient, albeit sub-optimal care is provided to the greatest number of patients. When supplies are radically insecure, providing a level of care ensures continued supply of resources for patients until replacement occurs. This consideration will need to be balanced against other considerations: if differential increases in some patient dosages frees up a scarce resource—if, for example, a high dose of a life saving drug given to some patients will free up beds in an overloaded hospital it may be, all other things being equal, justified to give small number of patients higher doses of a scarce drug to benefit a large set of other patients. As a baseline, however, in times of extreme scarcity and insecurity, scarce medical resources should be allocated in a way that maximizes the length of time a patient population can continue to receive clinically meaningful care.

## Risks from armed conflict

Armed conflict generates substantial risks for patients. First, civilians attempting to reach medical centers or hospitals may have to travel through active combat zones. Second, medical centers—and healthcare workers—may be targeted as strategic resources, sources of supplies, or in reprisal for perceived support of the opposition [12]. In October 2015, a hospital in Kunduz, Afghanistan, was allegedly hit by a US airstrike targeted using mistaken intelligence [13]. Even worse, health care facilities and providers in Syria may be deliberately targeted for looting or reprisals by armed groups [14, 15].

These risks may at times outweigh the benefits of clinical care in a way that, say, the risks a patient takes in a major US city in getting to a clinic for treatment do not. While there is always a small but nontrivial risk involved in transport for clinical care, in peacetime scenarios this is arguably always outweighed by the benefits of clinical care. In some cases, the risks of open movement in armed conflict may outweigh the benefits of clinical care. Moreover, healthcare workers themselves are scarce resources during armed conflict [8, 16]. Providing guidance to patients to reduce time in care facilities, where communication infrastructure is not so broken as to make such communication counterproductive [17], will help maintain supplies for a community and protect patients against a range of risks beyond those of their care.

## Culture

Armed conflicts are profoundly cultural events. The majority of modern armed conflicts are sectarian and/or civil in nature, rather than the international conflicts that defined the early twentieth century—15% of the world’s nations, it is believed, were involved in internal armed conflicts in 2009, though this rate is projected to decrease [18]. These conflicts often involve considerable ethnic or cultural conflict—arguably the most infamous being that of the Hutu and Tutsi peoples that led ultimately to the Rwandan Genocide in 1994. In these contexts, the provision of aid is often a flashpoint for violence if one group feels that healthcare workers are unfairly treating them, or, as clinical centers become strategic resources in their own right.

Second, warzones—and acute public health emergencies such as the recent Ebola virus disease outbreak in Western Africa—may require an international response to resolve. In this sense, culture becomes an important feature of clinical and public health practice when healthcare workers are from distinct cultural and ethnic groups to their patients [19]. The practice of healthcare by expatriate clinical staff can be fraught, particularly if they are introduced into cultural systems that are divergent from their own. In this context allocation paradigms, much like the conduct of clinical research in other nations [20] should be pursued in concert with local communities, rather than imposed upon them.

The particular features of armed conflict serve to modify existing principles of allocation. The idea of modification, here, is significant: it isn’t clear that we need *de novo* principles of allocation for armed conflict (or indeed any other disruptive event) [21]. Rather, the above considerations can serve to modify existing allocation principles to account for considerations that may not be relevant during peacetime, or may be outweighed by other ethical principles.

Here, we can reach some preliminary general conclusions about the ethics of allocation of scarce medical resources to noncombatant civilians during armed conflict. First, when considering the risks and benefits of care, the nontrivial risks of transport to or from, or residence at care facilities must be taken into account. Second, radical insecurity of resources mean that there is a *pro tanto* reason to restrict the use of nonperishable, continuous resources to a de minimis standard of clinical care, so that the harm of interrupted access to resources can be mitigated. Third and finally, sensitivity to cultural diversity is an essential element to caregiving, given the consequences incumbent on healthcare—and healthcare providers—when social cohesion is strained beyond breaking point.

## Hemodialysis in Syria: a test case for allocating scarce civilian medical resources during armed conflicts

To illustrate how these general conclusions might be resolved in practice, we consider the allocation of hemodialysis units in Syria, where seven years of armed conflict has led to hundreds of thousands of deaths and the displacement of approximately half the Syrian population. An underappreciated effect of the Syrian conflict has been the collapse of support for individuals receiving long-term clinical care, including patients with ESRD. We chose the example of ESRD because of its high cost, complex requirements of its provision, and imminent death when its treatment with renal replacement therapy is interrupted. Dialysis centers in areas such as Aleppo, Homs, and Idlib have been destroyed, looted, or occupied by armed groups [22].

In light of the above considerations of resource modalities, the risks of armed conflict, and culture, we offer the following principles for allocating hemodialysis sessions.

(1) *Balancing safety and supply* Hemodialysis should only be allocated when it is necessary to maintain patient outcomes. Given that replacement of supplies is far from guaranteed, and care at a facility risks healthcare workers and patients, the lowest possible frequency of hemodialysis cycles should be pursued, and the remaining supplies secured against future shortages. Even in resource-rich settings, reducing dialysis frequency is being considered as a safe and cost-effective approach under some conditions [23].

Urinary volume, as a proxy for residual renal function could be used as a guide to deciding which patient is a candidate for reduced dialysis frequency [24]. We recommend longer dialysis duration of about 5–6 h in patients who end up receiving a sub-optimal dialysis frequency. The longer duration partially compensates for the reduced frequency, does not incur additional transportation cost or risk, and requires little additional dialysis supplies.

Where possible, alternative modalities or relocation should be used to decrease stress on remaining supplies. Peritoneal dialysis using available catheters and home made solutions has been used save lives in acute kidney injury in resource-limited settings [25]. In the absence of hemodialysis supplies the same technique could be used in ESRD to buy time while waiting for new lines of supplies. If feasible, hemodialysis patients should be moved and housed in centers outside the conflict zone.

At a certain point the relationship between dose and frequency of dialysis, generally speaking, results in a sharp increase in risk for patients. This relationship is dependent on a patient’s remaining kidney function, reserve of other organs, and diet, but in general risk sharply increases if dialysis frequency falls below two sessions per week. Consider a recent comparison of outcomes between two dialysis facilities in Syria, one is in a besieged area and the other with access to supplies. One of the striking differences between the clinics was the frequency of dialysis: twice a week in the non-besieged facility and once a week in the besieged. After one year almost a half of the patients in the first unit died compared to 21% in the latter [26].

While there were surely other factors distinguishing the two facilities, the divergence of clinical outcomes based on a change in frequency of dialysis highlights the need for care providers to carefully select the rate of dialysis to balance patient safety against continuing supply. The increased risk to patients is not an in principle reason to refrain from reducing the frequency of dialysis. Increasing dialysis frequency and ultimately running out of supplies for all patients also entails risk, as does maintaining frequency while reducing the set of patients who receive care. Caregivers should strive to maintain sufficient quality of life for as wide a group of patients as possible.

(2) *Priority setting* Patients with ESRD are particularly vulnerable during armed conflicts: their expected comorbidities and lack of access to food and water render them in dire need of care. However, standard allocation principles (Table 1) are not sufficient in the face of this need. A lottery could be disastrous, as the distribution of already scarce dialysis resources would arguably lead to significant mortality in depriving patients of dialysis appropriate their prognosis, without necessarily adding value to those who do receive dialysis beyond their necessary courses.

We can envisage some kind of de facto first come, first served principle applying alongside others, in cases where uncertainty about the ability to travel make scheduling patients very difficult. That is, clinicians may want to set patients on courses of hemodialysis in cases where there is no guarantee that another patient will show up at the right time (say, because conditions of conflict have changed). We strongly discourage clinicians for adopting first come, first serve, however, as a principle for ex ante allocating resources to patients undergoing hemodialysis.

A tension arises between favoring the sickest, and maximizing life years for younger patients. On the face of it, emergencies such as severe hyperkalemia and volume overload should be given priority including additional dialysis sessions and even hospitalization with emergency dialysis. Relying solely on this principle, however, is not sufficient because it will eventually lead to a patient’s death of other uremic manifestations. Moreover, elderly patients tend to have increased co-morbidities and performing dialysis may provide little or no benefit. While the concept of palliative or conservative care in elderly patients with CKD is discussed as a futility question [27], its implementation has justice implications in the context of the Syrian conflict because it reduces spending of insecure resources.

In cases where supplies are not sufficient to ensure patient outcomes, hemodialysis should be prioritized according to

- A patient’s capacity to contribute to the care of others or provision of critical services to civilians during the conflict;
- The patient’s overall prognosis;
- A patient’s capacity to endure suboptimal clinical outcomes due to shortages.

That is, in conditions of scarce and insecure medical resources, those who can contribute to maintaining community support during the conflict-and potentially to later reconstruction efforts—should be prioritized. Then, we should allocate to those with the best overall prognosis. Finally, we should allocate to those whose suffering would be most acute were they to forgo dialysis.

The rationale for this is as follows. In conflict zones, maintaining essential services is vital not just to ESRD patients, but to everyone—utilitarian and social usefulness (itself promoting utility) principles outweigh other principles in a general sense, given the protracted nature of the conflict. Those who are both most likely to survive and contribute to caregiving in the wider community ought to be prioritized—in particular, those who care for other vulnerable individuals, such as young children or injured civilians, should be prioritized. Finally, those in the worst condition should be given care in order to relieve their suffering.

(3) *Alternative modalities* Peritoneal dialysis as an initial form of renal replacement therapy offers the advantage of eliminating the need for patient transportation in dangerous conditions and is less technologically demanding: there is no need for electricity when peritoneal dialysis is performed manually. The penetration of this modality in the management of ESRD patients in many developing countries, including Syria, has been low. One of the main reasons for this phenomenon is that, compared to developed countries, peritoneal dialysis in some developing countries is more expensive than hemodialysis [28].

Conservative non-dialytic management of elderly patients with advanced chronic kidney disease is an option that has been increasingly applied. The care is mostly palliative and patients may survive for months while clinically uremic due to some residual renal function [27].

(4) *Palliative care* The ultimate consequence of allocation paradigms, in any situation, is that a patient will inevitably be denied care. In these cases palliation for pain or other effects of forgoing dialysis should be, where possible, reserved for those who are most likely to be denied hemodialysis. Details of aspects of palliative renal care are beyond the scope of this work, but have been covered elsewhere [29].

Local religious figures ought to, where possible, be sourced to provide compassionate care—while there is some evidence that Islamic bioethical principles are consonant with standard Western accounts of ethical allocation principles, their framing may differ, and religious figures may be best placed to facilitate discussions about the allocation and timing of hemodialysis [30].

## Conclusion

Armed conflict presents unique challenges for allocation of hemodialysis. Seeking and providing medical care carries serious risks; medical resources are both scarce and uncertain. In these situations, allocation of these resources should proceed in such a way as to maintain patient health, preserve safety for patients and providers, and ration resources according to discrete priorities.

Ultimately, patients with complex medical needs, such as ESRD are disproportionately affected by armed conflict. The international community ought to lobby for improved resources for treating these patients, better access to hemodialysis centers, and for increased protections for clinics in areas of ongoing conflict. Until these broader changes occur, however, the aforementioned principles of resource allocation should guide clinician behavior.
